# Genetic Diversity, Antimicrobial Resistance Pattern, and Biofilm Formation in *Klebsiella pneumoniae* Isolated from Patients with Coronavirus Disease 2019 (COVID-19) and Ventilator-Associated Pneumonia

**DOI:** 10.1155/2021/2347872

**Published:** 2021-12-24

**Authors:** Asma Ghanizadeh, Maede Najafizade, Somaye Rashki, Zeynab Marzhoseyni, Mitra Motallebi

**Affiliations:** ^1^Department of Infectious Disease, School of Medicine, Kashan University of Medical Sciences, Kashan, Iran; ^2^Department of Immunology and Microbiology, Faculty of Medicine, Kashan University of Medical Sciences, Kashan, Iran; ^3^Infectious Diseases Research Center, Kashan University of Medical Sciences, Kashan, Iran

## Abstract

**Introduction:**

Patients with acute respiratory distress syndrome caused by coronavirus disease 2019 (COVID-19) are at risk for superadded infections, especially infections caused by multidrug resistant (MDR) pathogens. Before the COVID-19 pandemic, the prevalence of MDR infections, including infections caused by MDR *Klebsiella pneumoniae* (*K. pneumoniae*), was very high in Iran. This study is aimed at assessing the genetic diversity, antimicrobial resistance pattern, and biofilm formation in *K. pneumoniae* isolates obtained from patients with COVID-19 and ventilator-associated pneumonia (VAP) hospitalized in an intensive care unit (ICU) in Iran.

**Methods:**

In this cross-sectional study, seventy *K. pneumoniae* isolates were obtained from seventy patients with COVID-19 hospitalized in the ICU of Shahid Beheshti hospital, Kashan, Iran, from May to September, 2020. *K. pneumoniae* was detected through the *ureD* gene. Antimicrobial susceptibility testing was done using the Kirby-Bauer disc diffusion method, and biofilm was detected using the microtiter plate assay method. Genetic diversity was also analyzed through polymerase chain reaction based on enterobacterial repetitive intergenic consensus (ERIC-PCR). The BioNumerics software (v. 8.0, Applied Maths, Belgium) was used for analyzing the data and drawing dendrogram and minimum spanning tree. *Findings*. *K. pneumoniae* isolates had varying levels of resistance to antibiotics meropenem (80.4%), cefepime-aztreonam-piperacillin/tazobactam (70%), tobramycin (61.4%), ciprofloxacin (57.7%), gentamicin (55.7%), and imipenem (50%). Around 77.14% of isolates were MDR, and 42.8% of them formed biofilm. Genetic diversity analysis revealed 28 genotypes (E1–E28) and 74.28% of isolates were grouped into ten clusters (i.e., clusters A–J). Clusters were further categorized into three major clusters, i.e., clusters E, H, and J. Antimicrobial resistance to meropenem, tobramycin, gentamicin, and ciprofloxacin in cluster J was significantly higher than cluster H, denoting significant relationship between ERIC clusters and antimicrobial resistance. However, there was no significant difference among major clusters E, H, and J respecting biofilm formation.

**Conclusion:**

*K. pneumoniae* isolates obtained from patients with COVID-19 have high antimicrobial resistance, and 44.2% of them have genetic similarity and can be clustered in three major clusters. There is a significant difference among clusters respecting antimicrobial resistance.

## 1. Introduction

Coronavirus disease 2019 (COVID-19) is a new emerging disease in human population. The World Health Organization introduced COVID-19 as a pandemic in March 11, 2020. The disease is caused by a virus called severe acute respiratory syndrome coronavirus 2 (SARS-CoV-2) [1, 2]. The clinical manifestations of COVID-19 include fever, leukocytosis, severe hypoxemia, bilateral pulmonary infiltrates, multisystem inflammatory syndrome, and multiorgan failure. Five waves of COVID-19 outbreak have been reported in Iran so far [1].

The SARS-CoV-2 virus often affects ciliated cells in the alveolar epithelium and reduces their normal activities such as airway clearance. Subsequent gradual accumulation of fluid and residuals in the lung results in acute respiratory distress syndrome. Some afflicted patients need hospitalization in intensive care unit (ICU) to receive intensive care and mechanical ventilation [3].

Pulmonary infiltration and mechanical ventilation predispose patients with COVID-19 in ICU to secondary bacterial infections. Previous studies reported that 40%–86% of patients with COVID-19 receiving mechanical ventilation developed ventilator-associated pneumonia (VAP). The prevalence of VAP among patients in ICU varies according to their clinical conditions, ICU admission policies, and types of treatment [1, 4]. The most common gram-negative microorganisms contributing to VAP are Escherichia *coli*, *Klebsiella pneumoniae*, *Pseudomonas aeruginosa*, and *Acinetobacter baumannii* [2]. However, there is limited information about microorganisms which cause VAP among patients with COVID-19 in ICU [5].

*Klebsiella pneumoniae* (*K. pneumoniae*) is one of the most common pathogens contributing to VAP in ICU in the United States and Middle East countries like Iran [6]. It is a highly prevalent gram-negative bacterium which causes lethal nosocomial infections throughout the world [7]. In recent years, nosocomial infections caused by multidrug resistant (MDR) strains of *K. pneumoniae* (MDRKp) have been a major public health concern. Several studies reported the high prevalence of nosocomial infections caused by *K. pneumoniae* strains which were resistant to third generation cephalosporins, aminoglycosides, and quinolones. Some MDRKp strains have changed to extensive drug resistance (XDR) strains. These strains are usually a major threat to patients with serious health conditions such as COVID-19 due to the ineffectiveness of treatments against them and their high mortality rate [8–10].

*K. pneumoniae* can form biofilm which is an extracellular matrix consisted of proteins, exopolysaccharides, deoxyribonucleic acid (DNA), and lipopeptides and protects bacteria against antibiotics [11, 12]. Biofilm facilitates bacterial attachment to living and nonliving surfaces, prevents the penetration of antibiotics, and reduces the effects of antibiotics [13]. Thick biofilm formation is also observed in VAP and among colonized bacteria in endotracheal tube and ventilator circuit. These bacteria are mostly resistant to antibiotics due to their physical isolation from blood circulation, their ability to easily adapt to oxygen deprivation and pH changes, and inability of antibiotics to penetrate the full depth of the biofilm [14].

Moreover, *K. pneumoniae* has some virulence factors, such as capsular polysaccharides, lipopolysaccharides, types 1 and 3 fimbriae, outer membrane proteins, and factors determining iron acquisition and nitrogen source, and uses them for survival and evasion from the immune system of the body [15, 16]. Adequate knowledge about biofilm and virulence factors is critical for the effective management of nosocomial infections [17].

Bacteria have different genetic characteristics which determine their virulence and behaviors. Determining the genetic diversity of bacteria helps determine the most prevalent bacterial strains in a given setting, determine the sources of infections, and determine the best preventive measures and infection control policies for that setting [18, 19]. Typing methods are usually used for determining genetic diversity, assessing epidemiologic concordance, and determining the sources of infection. However, the reproducibility, stability, discriminatory power, and epidemiologic concordance of these methods should be assessed before their use [20].

Enterobacterial repetitive intergenic consensus (ERIC) is one of the repetitive elements which vary in different bacterial genomes in terms of pattern and number. Polymerase chain reaction (PCR) based on ERIC (ERIC-PCR) has successfully been used for the genotyping, strain diversity assessment, population analysis, and epidemiological assessments of different microbial pathogens and determining their taxonomy and phylogenetic relatedness [21]. Compared with other typing methods such as ribotyping, Pulse Field Gel Electrophoresis, and Multilocus Sequence Typing, ERIC-PCR is considered as a faster, more reliable, and more cost-effective technique for the molecular typing and the genetic diversity assessment of the *Enterobacteriaceae* family [22].

Kashan is a city in the center of Iran. Before the onset of the COVID-19 pandemic, high prevalence of MDRKp infections had been reported in the leading hospital of the city [23]. However, there were limited data about the microbiological characteristics of *K. pneumoniae* among patients with COVID-19 in the ICU of this hospital. The present study was conducted to narrow this gap. The study is aimed at assessing the genetic diversity, antimicrobial resistance pattern, and biofilm formation in *K. pneumoniae* isolates obtained from patients with COVID-19 and VAP in ICU.

## 2. Materials and Methods

### 2.1. Design

This descriptive study was conducted during the second wave of COVID-19 in Iran, i.e., from May to September, 2020.

### 2.2. Sample Collection

Seventy *K. pneumoniae* isolates were obtained from seventy patients with COVID-19 hospitalized in the ICU of Shahid Beheshti hospital, Kashan, Iran. This hospital is the main COVID-19 care center in Kashan city in the center of Iran. All *K. pneumoniae* isolates were obtained through sampling from patients' tracheal secretions and then were immediately cultured in an appropriate culture medium in the laboratory of the Microbiology Department of Kashan University of Medical Sciences, Kashan, Iran. Data on participants' demographic characteristics were collected from their medical records.

### 2.3. Ethical Considerations

The Ethics Committee of Kashan University of Medical Sciences, Kashan, Iran, approved this study (code: IR.KAUMS.MEDNT.REC.1399.034). Sampling and data collection were performed under the supervision of this committee. All participants provided written informed consent.

### 2.4. *K. pneumoniae* Detection

Phenotypic detection of *K. pneumoniae* to the species level was performed based on biochemical reactions, including reaction on SH2/indole/motility (SIM) medium, triple sugar iron (TSI) agar, urease production on urea agar, growth on Simmons' citrate agar medium, methyl red/Vogues-Proskauer (MR/VP), and ornithine decarboxylase (OD) test [23]. Before analysis, all isolates were cultured on brain-heart infusion agar (CONDA, Spain) and stored in tryptic soy broth (TSB) (CONDA, Spain) with 15% glycerol at a temperature of –70°C. Then, the PCR method was used to confirm *K. pneumoniae* isolates through detecting the *ure*D gene. This gene is responsible for urea hydrolysis. The forward primer 5′-CCCGTTTTACCCGGAAGAAG-3′ and the reverse primer 5′-GGAAAGAAGATGGCATCCTGC-3′ were employed for the amplification of 243 base pairs (bp) of the *ure*D gene [24]. PCR was performed in a final reaction volume of 25 *μ*L as follows: initial denaturation at 95°C for three minutes, denaturation at 95°C for thirty thirty-second cycles, annealing at 95°C for 45 seconds, elongation at 72°C for sixty seconds, and final extension at 72°C for sixty seconds. The final products of PCR were electrophoresed on agarose gel.

### 2.5. Antimicrobial Susceptibility Testing

Antimicrobial susceptibility testing was performed for all detected *K. pneumoniae* isolates using the Kirby-Bauer disc diffusion method based on the guidelines of the Clinical and Laboratory Standards Institute (CLSI) [25]. Accordingly, the antimicrobial resistance pattern of *K. pneumoniae* isolates was identified with respect to the following ten antibiotics: piperacillin/tazobactam (PTZ, 100/10 *μ*g), cefepime (CPM, 30 *μ*g), imipenem (IMP, 10 *μ*g), meropenem (Mer, 10 *μ*g), gentamicin (GM, 10 *μ*g), tobramycin (TOB, 10 *μ*g), aztreonam (AZT, 30 *μ*g), ciprofloxacin (CIP, 5 *μ*g), polymyxin B (300 *μ*g), and colistin (CST, 10 *μ*g). *K. pneumoniae* isolates which were resistant to three or more antimicrobial categories were considered as MDR [26]. Escherichia *coli* ATCC 25922 was used as control.

### 2.6. Biofilm Detection Using the Microtiter Plate Assay (MTP) Method

Biofilm was detected through the MTP method. MTP is a quantitative method to detect biofilm using a microplate reader. An overnight culture in the TSB culture medium was performed for each isolate at a temperature of 37°C. Then, a bacterial suspension in TSB was prepared and adjusted to 0.5 McFarland (1.5 × 10^8^ cfu/ml). This suspension was tenfold diluted to the concentration of 5 × 10^5^ (cfu/ml), and then, 200 *μ*L bacterial suspension was inoculated into 96-well flat-bottomed sterile microplate. Negative control wells containing 200 *μ*L TSB were also included in each test. Incubation at a temperature of 37°C was performed for 24 hours. After that, wells were gently washed three times using 200 *μ*L of distilled water, dried, and fixed using 99% methanol during twenty minutes. Thereafter, biofilm mass was stained for fifteen minutes using 200 *μ*L of 0.1% crystal violet dye. After air drying the wells, the dye of the biofilm which had lined the walls of the microplate was resolubilized using 5% isopropanol acid. Finally, the microplate was spectrophotometrically measured using a microplate reader at a wave length of 570 nm [27]. The optical density cutoff (ODc) was assigned as “an average OD of negative controls + (3 × standard deviation of negative controls).” Isolates were categorized respecting biofilm formation as follows: isolates with an OD equal to or less than ODc were considered as having no biofilm formation; isolates with an OD between ODc and 2ODc were categorized as weak biofilm producer; isolates with an OD between 2ODc and 4ODc were categorized as moderate biofilm producer; isolates with an OD more than 4OD were categorized as strong biofilm producer [27]. Each test was performed three times. In biofilm detection through the MTP method, PAO1 was used as positive control and TSB with 1% glucose was used as negative control.

### 2.7. ERIC-PCR

For epidemiologic typing, genomic DNA was extracted from bacterial cells through the method proposed by Purighalla et al. [28], and then, ERIC-PCR was performed for assessing genetic similarity among bacterial isolates. ERIC-PCR reactions were performed as previously described by Veresalovi et al. [29] using the primer ERIC1R (5′-ATG TAA GCT CCT GGG GAT TCAC-3′) and the primer ERIC2 (5′-AAG TAA GTG ACT GGG GTG AGC G-3′) (Metabion, Germany). PCR amplification was done using a mixture of 18 *μ*l of sterile distilled water, 2.5 *μ*l of 10× PCR buffer, 1 *μ*l of 10 molar dNTP, 1 *μ*l of each primer, 0.5 *μ*l of Taq polymerase, and 1 *μ*l of template DNA, i.e., a total volume of 25 *μ*l per reaction. PCR reaction consisted of an initial denaturation at 95°C for three minutes and then 35 thermal cycles consisting of denaturation at 94°C for one minute, annealing at 48°C for one minute, and final extension at 72°C for two minutes and at 72°C for five minutes.

### 2.8. ERIC Analysis

**Figure 1:**
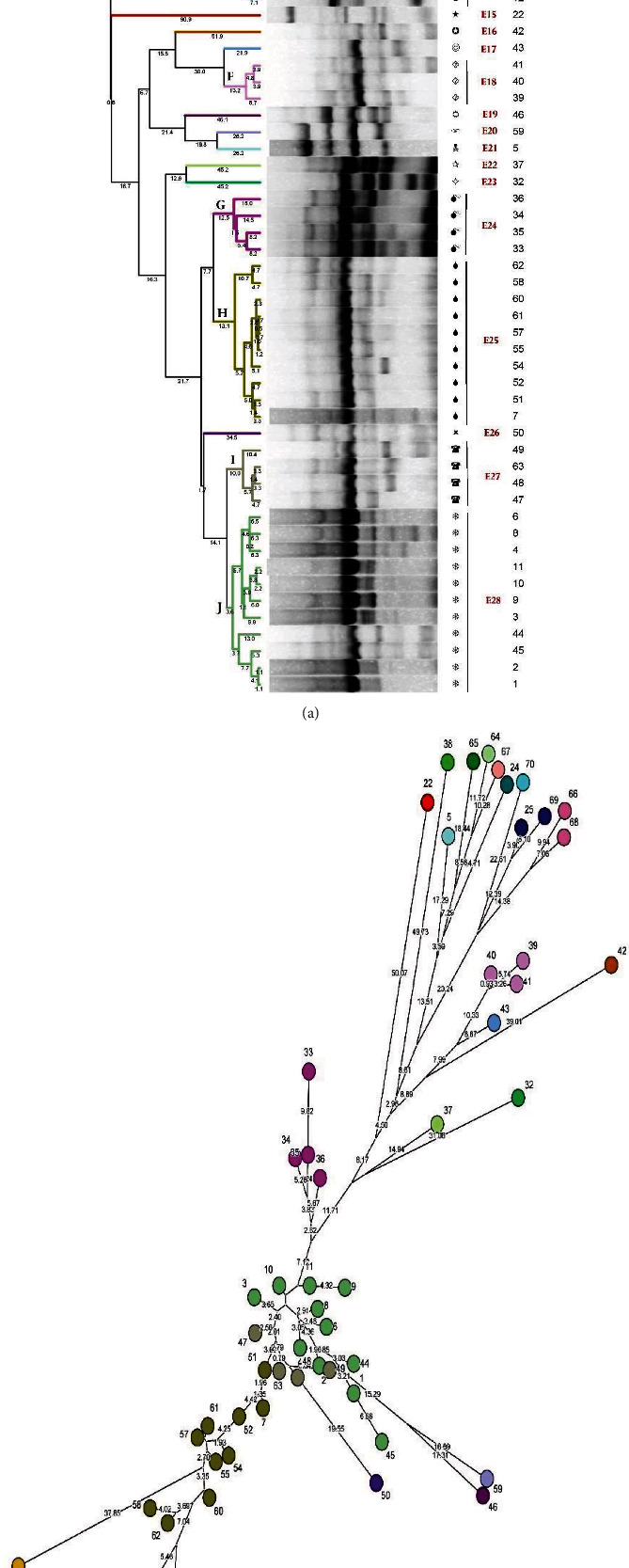
(a) Dendrogram. *K. pneumoniae* isolates with a genetic similarity of at least 80% were grouped into ten ERIC types, namely, A–J. (b) MST. Clusters were determined using the UPGMA method and Dice similarity coefficient. Numbers at the tip of each branch represent isolate numbers, and numbers over the lines show genetic difference between the isolates.

The BioNumerics software (v. 8.0, Applied Maths, Belgium) was used for band profile analysis. This version of the software has the options for analyzing and interpreting phenotypic and genotypic data such as the data obtained in gel- and sequence-based typing methods. For dendrogram construction, genetic similarity analysis was performed using Unweighted Pair Group Mean Method with Arithmetic mean (UPGMA), Dice similarity coefficient, and 1% band position tolerance. Only bands which sized 100–3000 bp according to the ladder were considered for analysis. The BioNumerics software creates groups with specific similarity range. The difference among band patterns was depicted using numbers over lines in dendrogram (Figure 1(a)) or numbers between isolates in minimum spanning tree (MST) (Figure 1(b)). Based on band analysis, isolates with a genetic similarity of 80% or greater were grouped into an ERIC type, while isolates without such similarity were considered as separate one-isolate ERIC type. ERIC types were named E1–En. The criteria for ERIC clustering in the BioNumerics software were a difference of 20% or less and the presence of more than one isolate in each cluster (A–J). Finally, MST was generated in the software (Figure 1(b)).

## 3. Statistical Analysis

Data were analyzed using the Fisher's exact and the Chi-square tests. *P* values less than 0.05 were considered statistically significant. Data analysis was performed using the SPSS software (v. 16.0).

## 4. Findings

In total, seventy tracheal *K. pneumoniae* isolates were obtained from patients with COVID-19 and VAP in ICU. Thirty-six isolates were from male patients, and 34 were from female patients. The mean of patients' age was 65.7 ± 10.5 years (Table 1).

Antimicrobial susceptibility testing showed that *K. pneumoniae* isolates had varying levels of resistance to antibiotics Mer (80.4%), CPM-AZT-PTZ (70%), TOB (61.4%), CIP (57.7%), GM (55.7%), and IMI (50%). Table 1 shows antimicrobial resistance pattern of *K. pneumoniae* for ten tested antibiotics. All *K. pneumoniae* isolates were sensitive to polymyxin B and colistin, while 77.14% of isolates were MDRKp.

Findings showed that 42.8% of *K. pneumoniae* isolates formed biofilm. Biofilm analysis showed that 2.8% of isolates were strong biofilm producer (*n* = 2), 31.4% of them were moderate biofilm producer (*n* = 22), 8.5% of them were weak biofilm producer (*n* = 6), and the remaining 57.1% of them did not form biofilm (*n* = 40) (Table 1).

Genetic similarity analysis through ERIC-PCR revealed 28 genotypes among the seventy *K. pneumoniae* isolates (E1–E28). These ERIC types consisted of ten genotypes with a genetic similarity of more than 80% and eighteen isolates with a genetic similarity of less than 80%. Each of these eighteen isolates was placed in a separate ERIC type (Figure 1(a)). During MST and dendrogram analysis, most isolates (*n* = 52) were placed in ten clusters (i.e., clusters A–J). The clusters were further categorized into three major clusters, i.e., clusters E, H, and J, with ten, ten, and eleven isolates, respectively. Moreover, 21 isolates were placed in seven minor clusters, namely, clusters A, B, C, D, F, G, and I, each with 2–4 isolates (Figure 1).

The analysis of antimicrobial resistance pattern showed that the most common patterns among *K. pneumoniae* isolates were, respectively, Mer+CPM+TOB+AZT+IMI+GM+CIP+PTZ and Mer+CPM+AZT+IMI+GM+CIP+PTZ, while the most common patterns in the three major clusters of E, H, and J were, respectively, Mer+CPM+TOB+AZT+IMI+GM+CIP+PTZ, Mer+TOB+CIP, and Mer+CPM+AZT+IMI+GM+CIP+PTZ. Comparing the results of antimicrobial susceptibility tests in the major clusters E and J showed that antimicrobial resistance to Mer, TOB, CIP, and GM in cluster J was significantly greater than cluster E (*P* < 0.05). Moreover, antimicrobial resistance to TOB in cluster H was significantly greater than cluster E. However, there was no significant difference between clusters J and H respecting antimicrobial resistance (Table 2). In molecular genotyping, there was no correlation between ERIC types and biofilm formation ability.

## 5. Discussion

This study assessed the genetic diversity, antimicrobial resistance pattern, and biofilm formation in *K. pneumoniae* isolates obtained from patients with COVID-19 and VAP in ICU. Most *K. pneumoniae* isolates in this study showed MDR patterns (77.14%) and were resistant to routinely used antibiotics for treating *K. pneumoniae* infections such as beta-lactams, aminoglycosides, and quinolones. Study results also revealed the high prevalence of antimicrobial resistance among *K. pneumoniae* isolates. The high prevalence of MDRKp infection in the present study was in line with the findings of previous studies [30, 31]. For example, two studies in Iran before the COVID-19 pandemic reported the high prevalence of *K. pneumoniae* among patients with VAP in ICU [23, 32]. MDR strains cause treatment failure and hence are associated with higher mortality rate than non-MDR strains among patients with COVID-19 in ICU. VAP caused by MDR pathogens can increase mortality rate among patients in ICU by 60% [9]. Many different factors can affect the emergence and spread of antimicrobial resistance, acquisition of antimicrobial resistance genes, and transmission of these genes. These factors include healthcare exposure (such as hospitalization), use of medical devices, shortage of diagnostic equipment, lack of efficient surveillance systems, immunosuppression, travel to areas with high endemicity of MDR bacteria, no use of new antimicrobial treatments, use of antibiotics in agriculture and animal food products, and extensive and inappropriate use of antibiotics in hospital wards [33]. These factors might also have contributed to the high prevalence of antimicrobial resistance in the present study. Inappropriate use of antibiotics is a leading cause of antimicrobial resistance. International reports show that despite the low risk of bacterial infection and no necessity of antibiotic therapy for mild to moderate cases of viral infections [34], around 70% of hospitalized patients with COVID-19 receive broad-spectrum antibiotics as prophylaxis [35].

The high prevalence of antimicrobial resistance to antibiotics Mer and CIP is an important finding because these were the most commonly used antibiotics in the study setting for the management of *K. pneumoniae* infections. As *K. pneumoniae* is a major cause of VAP in ICU, its resistance to these antibiotics makes treatment difficult and causes challenges for the management of VAP among patients with COVID-19 in ICU. The mechanisms of resistance to carbapenems such as Mer among *K. pneumoniae* include changes in membrane permeability, production of wide-spectrum beta-lactamases, changes in porins, and expression of the efflux system genes [36]. Moreover, studies showed that the most important mechanism of *K. pneumoniae* resistance to fluoroquinolones such as CIP is plasmid-mediated horizontal gene transfer [37, 38]. Our findings also revealed the high prevalence of resistance to GM among *K. pneumoniae* isolates. Previous studies also reported acquired resistance to aminoglycosides such as GM among both gram-negative and gram-positive bacteria [39, 40]. The three mechanisms of such resistance are changes in the ribosomal binding sites of antibiotics, reduced penetration of antibiotics into bacteria, and enzymatic inactivation of antibiotics. Enzymatic inactivation is the most prevalent mechanism of resistance to aminoglycosides [41].

Study findings also showed that 42.8% of *K. pneumoniae* isolates were able to form biofilm, and 34.2% of those with this ability were moderate to strong biofilm producers. Biofilm formation is an important step in the development and stabilization of opportunistic nosocomial VAP because it protects bacteria against phagocytosis. Colonization of VAP-induced bacteria in endotracheal tube and ventilator circuit is very common, and there is a well-known significant relationship between such colonization and nosocomial pneumonia [42]. *K. pneumoniae* is a major cause of VAP, and biofilm formation is an important pathogenic factor among patients with VAP induced by *K. pneumoniae*. Biofilm formation ability seems to have direct relationship with environmental resistance among *K. pneumoniae* strains [43]. A former study into the biofilm formation ability of *K. pneumoniae* reported a biofilm formation rate of 62.5% [44]. Another study found that 37.6% of *K. pneumoniae* strains formed biofilm [13]. This difference among studies regarding the rate of biofilm formation by *K. pneumoniae* is attributable to the differences in the geographical area, setting, and sample size of the studies. Effective management of infections caused by biofilm-producing microorganisms using available antibiotics is essential in healthcare settings. Recently, some novel biofilm-eliminating strategies have been developed. Examples of these strategies are use of bacteriophage, weak organic acids, and photo irradiation. Nonetheless, further studies are still needed to produce firmer evidence in this area [45].

MST and dendrogram analysis in this study revealed 28 different ERIC types, namely, ten prevalent types and eighteen unique types. This finding denotes the great diversity of the *K. pneumoniae* isolates obtained from the study setting. Several previous studies in Iran and other countries also reported genetic diversity among *K. pneumoniae* isolates. For instance, a study on CST-resistant *K. pneumoniae* isolates in the southwest of Iran reported 23 ERIC types among 26 isolates [46]. Another study reported 32 ERIC types among 35 K*. pneumoniae* isolates. Moreover, a study into the genetic relatedness of MDRKp isolates in hospitals in Egypt reported 21 ERIC types and great genetic diversity [19]. These contradictory findings are related to the high heterogeneity of pathogenic *K. pneumoniae* due to differences in its nucleotide sequences [47].

Study findings also showed that *K. pneumoniae* isolates in the largest cluster, i.e., cluster J, had high antimicrobial resistance to antibiotics Mer, CPM, TOB, AZT, GM, CIP, and PTZ, and the most prevalent antimicrobial resistance pattern among them was Mer+CPM+TOB+AZT+IMI+GM+CIP+PTZ. Antimicrobial resistance to Mer, TOB, GM, and CIP in this cluster was significantly higher than cluster H. Moreover, antimicrobial resistance to TOB in cluster J was significantly higher than cluster E. These findings denote significant relationship between ERIC clusters and antimicrobial resistance. However, there was no significant difference among the three major clusters E, H, and J respecting biofilm formation. The high diversity of *K. pneumoniae* isolates in the present study is an important finding because more than 70% of these isolates are MDR and can use mechanisms such as horizontal gene transfer to transfer this resistance to the bacteria which induce healthcare-associated infections. Different clones may have different antimicrobial resistance patterns and thereby can cause more difficulties in the treatment of their associated infections [22]. Given the significant role of antimicrobial resistance in the management of VAP, the high prevalence of resistant strains can complicate the conditions of patients with COVID-19. Therefore, determination of antibiotic-resistant bacteria, selection of appropriate antibiotics for VAP management, and close adherence to nosocomial infection management guidelines in hospital wards, particularly in ICU, are recommended to reduce mortality rate among these patients.

The rates of VAP between centers managing COVID-19 are likely to vary depending on the clinical characteristics of the patients managed, differential ICU admission policies, and clinical factors such as use of immunosuppressive therapies. However, it is thought that increasing the number of nurses in the ICU, educating nurses about oral hygiene, and head of bed elevation in these centers can help reduce VAP cases.

## 6. Conclusion

This study suggests that *K. pneumoniae* isolates obtained from patients with COVID-19 have high antimicrobial resistance, and the prevalence of MDR strains among them is very high (77%). Moreover, 42.8% of these isolates are biofilm producer, and 44.2% of them have genetic similarity and are clustered in three main clusters with significant differences among clusters respecting genetic similarity and antimicrobial resistance of the isolates. Determining the characteristics of these isolates helps medical specialists more effectively manage their associated infections and use more effective infection management policies for each setting. Further studies are recommended for assessing genetic diversity, ERIC types, and antimicrobial resistance pattern among different bacterial species which cause VAP among patients with COVID-19 in order to facilitate patient recovery. Future studies are needed to assess bacterial colonization among patients with COVID-19 at the time of their hospital admission.

## Figures and Tables

**Table 1:** Characteristics of patients and *K. pneumoniae* isolates.

No.	Patients		*K. pneumoniae* isolates				
	Gender	Age (year)	Antimicrobial resistance pattern	MDR	Biofilm formation	ERIC type	Cluster
1	Female	71	Mer, CPM, TOB, AZT, GM, CIP, PTZ	+	No	E28	J
2	Female	50	Mer, CPM, TOB, AZT, IMI, GM, CIP, PTZ	+	No	E28	J
3	Male	56	Mer, CPM, TOB, AZT, IMI, GM, CIP, PTZ	+	No	E28	J
4	Male	78	Mer, CPM, TOB, AZT, IMI, GM, CIP, PTZ	+	No	E28	J
5	Male	59	—	_	No	E21	—
6	Female	69	Mer, CPM, TOB, AZT, GM, CIP, PTZ	+	No	E28	J
7	Female	72	Mer	_	No	E25	H
8	Male	66	Mer, CPM, TOB, AZT, IMI, CIP, PTZ	+	No	E28	J
9	Female	49	Mer, CPM, AZT, CIP, PTZ	+	Moderate	E28	J
10	Female	81	Mer, CPM, TOB, AZT, IMI, GEM, CIP, PTZ	+	No	E28	J
11	Female	75	Mer, CPM, AZT, GM, CIP, PTZ	+	No	E28	J
12	Male	53	Mer, CPM, AZT, IMI, CIP, PTZ	+	Moderate	E14	E
13	Male	52	Mer, CPM, AZT, IMI, GM, CIP, PTZ	+	Weak	E14	E
14	Male	65	Mer, CPM, TOB, AZT, IMI, GM, CIP, PTZ	+	No	E14	E
15	Male	78	Mer, CPM, AZT, IMI, GM, CIP, PTZ	+	Weak	E14	E
16	Male	61	—	_	No	E14	E
17	Male	64	—	_	Moderate	E14	E
18	Female	80	Mer, CPM, AZT, IMI, GM, CIP, PTZ	+	No	E14	E
19	Female	79	Mer, CPM, TOB, AZT, IMI, GM, CIP, PTZ	+	No	E14	E
20	Male	73	—	_	Moderate	E14	E
21	Female	63	Mer, CPM	_	Moderate	E11	—
22	Female	46	Mer, CPM, AZT, IMI, CIP, PTZ	+	No	E15	—
23	Female	55	—	_	Moderate	E14	E
24	Male	58	—	_	Moderate	E5	—
25	Female	61	—	_	Moderate	E13	D
26	Male	51	Mer, CPM, TOB, AZT, IMI, GM, CIP, PTZ	+	No	E10	B
27	Male	76	Mer, CPM, TOB, AZT, IMI, GM, CIP, PTZ	+	No	E10	B
28	Male	79	Mer	_	Moderate	E10	B
29	Female	67	Mer, CPM, TOB, AZT, GM, PTZ	+	No	E10	B
30	Female	58	CPM, TOB, AZT, GM, CIP	+	No	E9	—
31	Female	59	TOB, CIP, PTZ	+	No	E8	A
32	Male	80	Mer, CPM, TOB, AZT, IMI, GM, CIP, PTZ	+	No	E23	—
33	Male	77	Mer, CPM, TOB, AZT, GM, CIP	+	No	E24	G
34	Male	49	Mer, CPM, TOB, AZT, IMI, GM, CIP, PTZ	+	Moderate	E24	G
35	Male	52	Mer, CPM, TOB, AZT, IMI, GM, CIP, PTZ	+	Strong	E24	G
36	Male	54	Mer, CPM, TOB, AZT, IMI, GM, CIP, PTZ	+	Moderate	E24	G
37	Male	73	Mer, CPM, TOB, AZT, IMI, GM, CIP, PTZ	+	No	E22	—
38	Male	67	Mer, CPM, TOB, AZT, GM, CIP, PTZ	+	Moderate	E1	—
39	Male	61	Mer, CPM, AZT, IMI, CIP, PTZ	+	Moderate	E18	F
40	Female	73	Mer	_	Weak	E18	F
41	Female	70	Mer	_	Moderate	E18	F
42	Female	84	PTZ	_	Weak	E16	—
43	Male	50	Mer, CPM, TOB, AZT, IMI, GM, CIP, PTZ	+	No	E17	—
44	Male	72	Mer, CPM, TOB, AZT, IMI, GM, CIP, PTZ	+	Moderate	E28	J
45	Female	74	Mer, TOB, GM, CIP	+	No	E28	J
46	Male	60	Mer, CPM, TOB, AZT, IMI, GM, CIP, PTZ	+	Moderate	E19	—
47	Female	64	Mer, CPM, TOB, AZT, IMI, GM, CIP, PTZ	+	Moderate	E27	I
48	Male	56	Mer, CPM, TOB, AZT, IMI, GM, CIP, PTZ	+	Moderate	E27	I
49	Male	55	Mer, CPM, AZT, IMI, GM, CIP, PTZ	+	No	E27	I
50	Male	71	Mer, TOB	_	Moderate	E26	—
51	Female	77	Mer, CPM, TOB, AZT, IMI, GM, CIP, PTZ	+	No	E25	H
52	Female	83	Mer, CPM, AZT, IMI, GM, CIP, PTZ	+	No	E25	H
53	Female	48	CPM, AZT, CIP, PTZ	+	No	E8	A
54	Female	74	Mer, CPM, TOB, IMI, CIP, PTZ	+	No	E25	H
55	Male	66	Mer, TOB, CIP	+	Moderate	E25	H
56	Male	58	Mer, CPM, TOB, AZT, CIP, PTZ	+	Weak	E7	—
57	Female	68	Mer, TOB, CIP	+	No	E25	H
58	Female	69	Mer, CPM, TOB, AZT, IMI, GM, CIP, PTZ	+	No	E25	H
59	Male	84	Mer, CPM, TOB, AZT, GM, CIP	+	Moderate	E20	—
60	Female	79	CPM, AZT	_	No	E25	H
61	Male	70	Mer, CPM, AZT, CIP, PTZ	+	No	E25	H
62	Male	63	Mer, TOB, AZT, IMI, GM, CIP, PTZ	+	No	E25	H
63	Female	67	Mer, TOB, CIP, PTZ	+	No	E27	I
64	Female	81	Mer, CPM, TOB, AZT, PTZ	+	Strong	E4	—
65	Male	48	Mer, TOB, AZT, IMI, CIP, PTZ	+	Weak	E2	—
66	Male	62	Mer, CPM, TOB, AZT, IMI, GM, CIP, PTZ	+	No	E12	C
67	Male	79	Mer, CPM, TOB, AZT, GM, CIP, PTZ	+	No	E3	—
68	Female	73	Mer, CPM, TOB, AZT, IMI, GM, CIP, PTZ	+	No	E12	C
69	Female	71	Mer, CPM, AZT, IMI, GM, CIP, PTZ	+	No	E13	D
70	Female	65	TOB, CIP	_	Moderate	E6	—

**Table 2:** Comparing the three major clusters of *K. pneumoniae* isolates obtained from patients with COVID-19.

Cluster	Antibiotic							
	Mer	CPM	TOB	AZT	IMI	GEM	CIP	PTZ
J (*n* = 11) (%)	11 (100)	10 (90.9)	9 (81.8)	10 (90.9)	6 (54)	9 (81.8)	11 (100)	10 (90.9)
H (*n* = 10) (%)	9 (90)	5 (50)	6 (60)	6 (60)	5 (50)	4 (40)	8 (80)	6 (60)
E (*n* = 10) (%)	6 (60)	6 (60)	0 (0)	6 (60)	6 (60)	0 (0)	6 (60)	6 (60)

*P* value	0.4762^#^	0.635^#^	0.3615^#^	0.0635^#^	1^#^	0.0805^#^	0.2143^#^	0.1486^#^
	0.0351^^^	0.1486^^^	≤0.001^^^	0.0635^^^	1^^^	≤0.001^^^	0.0351^^^	0.1486^^^
	0.3034^∗^	1^∗^	0.0108^∗^	1^∗^	1^∗^	0.0902^∗^	0.6285^∗^	1^∗^

^#^Comparison between clusters J and H. ^^^Comparison between clusters J and E. ^∗^Comparison between clusters H and E.

## Data Availability

Data are available on request.
